# Between security and convenience: Facial recognition technology in the eyes of citizens in China, Germany, the United Kingdom, and the United States

**DOI:** 10.1177/09636625211001555

**Published:** 2021-03-26

**Authors:** Genia Kostka, Léa Steinacker, Miriam Meckel

**Affiliations:** Freie Universität, Berlin, Germany; University of St. Gallen, Switzerland; University of St. Gallen, Switzerland

**Keywords:** acceptance, facial recognition technology, privacy, public opinion, security

## Abstract

How does the public perceive facial recognition technology and how much do they accept facial recognition technology in different political contexts? Based on online surveys resembling the Internet-connected population in China, Germany, the United Kingdom, and the United States, our study finds that facial recognition technology enjoys generally highest acceptance among respondents in China, while acceptance is lowest in Germany, and the United Kingdom and the United States are in between. A closer examination through the lens of an integrated technology acceptance model reveals interesting variations in the selected four countries based, among other factors, on socio-demographic factors as well as perceived consequences, usefulness, and reliability of facial recognition technology. While previous research has pointed out that facial recognition technology is an instrument for state surveillance and control, this study shows that surveillance and control are not foremost on the minds of citizens in China, Germany, the United Kingdom, and the United States, but rather notions of convenience and improved security.

## 1. Introduction

Facial recognition technology (FRT)—which seeks to match people from a digital image or video with various identifying attributes—is increasingly commonplace (Burt, 2019) and the subject of increased debate. On one hand, FRT is seen as a potentially powerful instrument for law enforcement and commercial interests. Government applications of FRT are wide-ranging, including for improved security in schools and airports (Gillespie, 2019), location of missing people (Bernal, 2019), detection of rare diseases (Mjoseth, 2017), fighting against crime and corruption (Chen, 2019), paying out pensions (Zhan, 2019), imposing age restrictions on online viewing of pornography (O’Mallon, 2019), and limiting gambling addiction (Robson, 2011). At the same time, FRT poses ethical dilemmas, since the technology is subject to biases, remains prone to inaccuracies, and can intrude on privacy when used clandestinely. The promised benefits of FRT applications come with trade-offs regarding digital mass surveillance, discrimination, privacy intrusion, as well as infringement on human rights (Smith, 2018; West, 2019). As a multi-purpose tool, the same underlying technology thus helps people and save lives and can also horrify individuals and pose ethical conundrums (West, 2019).

Given the dystopian aspects of this technology, there is much discussion within governments, policy think-tanks and media about whether and how FRT should be deployed and whether stricter regulations or even bans are the appropriate response. Many governments welcome FRT and are investing heavily in applications of the technology. France, for instance, introduced a nationwide facial recognition ID program for public services in 2019 (Fouquet, 2019). More recently, the COVID-19 pandemic has spurred a rise in FRT applications to aid the response. Companies whose FRT algorithms recognize compliance with mask mandates (Yan, 2020), temperature scanning (Burt, 2020), or quarantine order (Deutsche Welle, 2020) offer to assist in the management of public health measures.

Meanwhile, some countries and, particularly, cities have taken the lead in imposing regulation and even outright bans of FRT targeting both public and private uses of FRT. The State of California, for instance, became the first state in the United States to ban use of FRT by law enforcement agencies in 2019 (Greene, 2019). In 2020, the city of Portland passed a ban of FRT not only for all city departments, including local police, but also for private retailers, such as hotels and restaurants (Metz, 2020).

While FRT is rapidly being rolled out, there is surprisingly little known about how citizens actually perceive FRT, whether opinions differ across countries, and most importantly, what factors appear to drive acceptance of the technology. Our article fills this gap with an online survey of 6633 citizens in China, Germany, the United Kingdom, and the United States conducted between August and September 2019. These countries were chosen because of a range of relevant factors. First, to ensure that FRT would be a relevant subject of study in each country, we chose the four nations with high rates of closed-circuit television (CCTV) cameras per 100 individuals: The United States leads the worldwide list of available data with a rate of 15.3 cameras, followed by China at 14.4, the United Kingdom at 7.5, and Germany at 6.3 (PreciseSecurity, 2019). At the same time, in terms of people’s general assessment of state-led public video documentation, the latest World Value Survey shows that the four selected countries differ: In China, 43% of participants believe that the government should “definitely” have the right “to keep people under video surveillance in public areas,” compared with 26% in Germany, 35% in the United Kingdom, and 23% in the United States (World Values Survey, 2020). In addition, our selection represents a politically diverse group including a one-party socialist republic, a federal parliamentary republic, a parliamentary constitutional monarchy, and a presidential republic. Comparing these nations might result in useful empirical differences in how citizens respond to FRT within their political context.

A number of models have been developed to assess the diverse influences on an individual’s propensity to accept—and be likely to use—a technology, for example, the technology acceptance model (TAM) and its extensions (TAM 2, TAM 3), and the unified theory of acceptance and use of technology (UTAUT) among others (Davis, 1989; Venkatesh and Davis, 2000; Venkatesh et al., 2003). However, these were by and large developed originally to assess acceptance of information technology on the job. Hence, some of the included aspects appear less relevant for an examination of FRT. A growing literature on the privacy-security trade-off (e.g. Davis and Silver, 2004) further provides insights on individuals’ willingness to adopt technologies. With regards to the acceptance of biometrics more broadly, the realm of trust and privacy literature offers the valuable insight that perceived consequences, particularly with regards to privacy risks, are an important driver of attitudes toward such technologies (Miltgen et al., 2013). Therefore, we construct a combined conceptual model that is technology-specific but can be applied to diverse country contexts.

Drawing on UTAUT and the privacy-security trade-off literature, our study investigates the effects of socio-demographic factors, experience, and perceived risks, benefits, usefulness, and reliability on public attitudes toward FRT. The survey participants resemble the Internet-connected population in the selected four countries and their responses are weighted by age, gender, and region. The objectives of this study are (1) to document the overall level of citizens’ (non-)acceptance of different types of FRT usage and (2) to identify underlying factors that are associated with variations in acceptance of FRT across the four countries based on an integrated model.

The study contributes to existing research in the realm of technology acceptance and privacy research in several ways. First, the unique survey dataset exploited here is the first online survey in these four countries about public opinion on FRT, allowing a comparison between different socio-economic and cultural contexts. While a growing number of studies have turned to the investigation of public opinion on FRT, the findings so far are based on single-country analysis, which makes it difficult to generate broader claims about public attitudes on FRT. Our study sheds new light on variation in FRT acceptance among countries and different citizen groups within each country. Second, by integrating aspects of different TAMs and adding the factor of risk perception from the privacy-security trade-off literature, our model offers a more comprehensive and technology-specific framework for the particular area of biometric technologies. The analysis shows how citizens’ socio-demographic background, experience with FRT, and their perception of the technology’s consequences, usefulness, and reliability shape citizens’ attitudes toward applications, expanding existing research on technology acceptance and usage (Venkatesh et al., 2003). Finally, by looking at both commercial and public types of FRT, we present an examination of acceptance of FRT in two vastly different contexts. We show that having volitional control over using FRT can influence attitudes toward the technology compared to non-optional usage. In the next section, we review the relevant theoretical and empirical literature to develop our integrated conceptual model of FRT acceptance.

## 2. Model development

### FRT and global adoption

The nature and frequency of FRT adoption by commercial companies and governments varies greatly. Fueled by the rise of smartphone cameras, commercially driven adoption of FRT has been rising globally. Looking at the four countries investigated in our study, major companies, such as Apple, Facebook, Google, Alibaba, Baidu, American Airlines, and Walmart have incorporated FRT for the purposes of customer recognition, security, intelligent marketing, and digital payment. While these firms collect vast amounts of data, FRT deployment by them has received less criticism than government installations. More recently, however, questions have arisen regarding the extent to which these companies protect the data they collect from consumers spurring debate about the necessary protections required prior to widespread adoption of FRT. These are particularly thorny questions in authoritarian countries, such as China, where the government keeps enterprise on a tight leash (Lv and Luo, 2018).

Government-run FRT systems are also spreading rapidly across countries, with more than 64 countries having rolled out some type of FRT scheme (Feldstein, 2019). Of the four countries under study here, China has most strongly embraced government applications of FRT, Germany the least, with the UK and the US governments in between (Authors, 2020). In China alone, it is estimated that more than 170 million closed-circuit television (CCTV) cameras were in use in 2018, with an additional 400 million to be installed by 2020 (BBC, 2018). While the Chinese state frames FRT as an effective tool to improve public service provision and supervise corrupt government officials (Jiangxi Government, 2019), recent evidence shows that the technology has darker uses including the tracking of Muslim Uighur minorities in Xinjiang province (Leibold, 2020). In the United States and Europe, the interest in deploying the technology for government uses is also high, but implementation has been slower (Prakash, 2018). The Federal Bureau of Investigation’s (FBI) facial recognition database currently includes 641 million images which can be searched anytime without an official warrant (Harwell, 2019). In the United Kingdom, major police departments utilize live face-tracking, a development condoned by a British High Court in a precedent-setting lawsuit regarding real-time uses of FRT (Satariano, 2019). In Germany, where the topic of data privacy is especially prominent in public debate, there is much controversy surrounding FRT (Fürstenau, 2019). As of 2019, major German airports offer the EasyPASS system with integrated FRT for identity verification. A pilot project at Berlin’s Südkreuz train station also tested FRT and generated significant negative blowback from data privacy advocates (Delcker, 2019). Given how swiftly both commercial and government uses of FRT are emerging, the question of what drives public opinion of this momentous socio-technological shift is highly relevant to ongoing debate about how FRT should and should not be woven into public life.

### Public attitudes toward FRT

Awareness of FRT as an underlying technology of various applications has been steadily growing, as worldwide Google trends show: While searches for “facial recognition” have increased since 2004, among the top 10 related search terms are “mobile app,” “emotional recognition,” “iPhone X,” “police,” and “artificial intelligence” (Google Trends, 2020). These growing query combinations indicate a consciousness of the technology. Coupled with the range of endogenous concerns about FRT—biases, inaccuracies, and privacy violations—the level of its public acceptance thus proves a timely subject of study.

Looking at the four countries investigated in our study, existing research points to varying public attitudes toward FRT as well as to similar biometric technologies. With regard to China, previous work has assessed public acceptance of surveillance technologies and social scoring systems (Ahmed, 2018; Kostka, 2019; Kostka and Antoine, 2020). In a study of 6100 Chinese citizens, 83% of respondents indicated that they would like to have more control over their data and 75% would prefer the option to have traditional methods of identification over FRT (The Nandu Personal Information Protection Research Center, 2019). Other studies show that Chinese citizens trust the central government more than private enterprises to manage and implement surveillance technologies (Kostka, 2019). Studies on public acceptance of FRT in Germany are scarce and mainly assessed German citizens’ views on surveillance technologies in general (Van Heek et al., 2017). Relatively more research exists on public opinion in the United Kingdom and the United States. A 2019 poll of 4109 adults in the United Kingdom finds that 77% of respondents are uncomfortable with FRT being deployed by commercial companies, 49% support FRT use for policing purposes given appropriate safeguards, while 67% oppose it in schools and 61% oppose its use on public transport (Ada Lovelace Institute, 2019). Another survey using convenience sampling with 282 UK participants explored attitudes toward biometrics analysis overall and found that UK respondents were uniformly more comfortable with their biometric data being held by a government than a private company (Buckley and Nurse, 2019). For the United States, a Pew Research Center survey of 4272 adults found that acceptance varies for different types of FRT, depending on who is using the technology. Out of all respondents, 56% trust law enforcement actors to employ FRT responsibly, while only 36% think the same of the technology when used by private companies and only 18% when used by advertisers (Smith, 2018). These studies point to international differences in public opinion and offer a starting point to derive hypotheses for factors that explain cross-country variation in FRT acceptance levels.

### Prior factors: Socio-demographics and experience

In existing research, including on privacy-security trade-off and the TAM and UTAUT models, findings are often inconclusive about how individual socio-demographic characteristics affect citizens’ technology acceptance as prior factors. A US survey on biometric security technologies (*n* = 410) finds that acceptance increases with higher income and education, while age and gender are not crucial factors (Fletcher et al., 2017). In contrast, a telephone survey with 2176 German citizens shows that respondents with lower education and women are more accepting of surveillance policies (Trüdinger and Steckermeier, 2017). According to a Pew Research Center study, acceptance of FRT increases with age: 67% of Americans above the age of 65 trust law enforcement with the technology, as opposed to 49% of Americans ages 18–29 (Smith, 2018). The same survey also finds race to be an important factor: about 60% of White Americans said they trust law enforcement with the technology, but only 43% of Black respondents did. In addition, research shows that living in an urban area or larger city affects citizens’ attitudes. As crime rates are much higher in bigger cities and urban areas (Glaeser and Sacerdote, 1999), one can assume that residents in urban locations have stronger preferences for additional security measures. Moreover, people living in rural areas or smaller cities might have less firsthand experience with FRT. For instance, people in rural areas are probably less likely to see surveillance cameras in their neighborhoods.

Furthermore, an individuals’ experience matters. Studies have shown that familiarity with a particular technology is positively associated with the adoption, usage, and acceptance of specific technologies (e.g. Idemudia and Raisinghani, 2014; Komiak and Benbasat, 2006). A survey with 282 UK participants on attitudes toward biometrics also finds that citizens are more accepting of technologies with which they are most familiar (Buckley and Nurse, 2019). Based on these studies, we derive the first two sets of hypotheses for our study: H1.1, H1.6 and H2.1, H2.3 (see Table 1).

**Table 1.** Measurements and hypotheses.

Category	Measurement	Hypothesis
Socio-demographic factors		
Age	*In years (open box)*	H1.1: FRT acceptance is higher among older citizens
Gender	*0* *=* *male, 1* *=* *female*	H1.2: FRT acceptance is higher among female citizens
Income	*Germany, UK, US: 1* *=* *Under 250, 2* *=* *250–500, 3* *=* *500–1000 . . . 12* *=* *more than 15,000, 99* *=* *Prefer not to say (in local currency)*; *China: 1* *=* *under 700, 2* *=* *700–1400, 3* *=* *1400 –2100 . . . 12* *=* *more than 28,000, 99* *=* *prefer not to say (in CNY);* *regrouped: 1* *=* *Low (1–3), 2* *=* *Medium (4–6), 3* *=* *High (7–12), 99* *=* *Prefer not to say (99)*	H1.3: FRT acceptance is higher among citizens with higher income
Education	*1* *=* *I don*’*t have formal education, 2* *=* *High school diploma or equivalent, 3* *=* *Vocational training, 4* *=* *Bachelor*’*s degree, 5* *=* *Master*’*s or Doctorate*’*s degree*	H1.4: FRT acceptance is higher among citizens with more education
Ethnic Group	*0* *=* *Minority, 1* *=* *Majority, 99* *=* *Don*’*t know, dummy variable created: 0* *=* *Majority/Don’t know, 1* *=* *Minority*	H1.5: FRT acceptance is higher among ethnic majority
Living in rural or urban area	*0* *=* *Rural, 1* *=* *City*	H1.6: FRT acceptance is higher among citizens living in urban areas
Experience		
Exposure to FRT	*Use occasions* 1 = smartphone use, 2 = smart devices or gadgets, 3 = public streets, 4 = railway, subway stations, 5 = customs control or security check at airports, 6 = tourist attractions, 7 = identity verification for financial matters, 8 = shopping malls, private shops, 9 = schools or universities, 10 = private households, 11 = others, 12 = none of the above	H2.1 FRT acceptance is higher among citizens who have been exposed to many instances of FRT
Frequency of FRT use	*Frequency in private use* 1 = Never, 2 = Several times in my life, 3 = Several times a year, 4 = Several times a month, 5 = Several times a week, 6 = Most days, 7 = Everyday *Frequency in public use* 1 = Never, 2 = Several times in my life, 3 = Several times a year, 4 = Several times a month, 5 = Several times a week, 6 = Most days, 7 = Everyday	H2.2: FRT acceptance is higher among citizens who have used FRT privately at higher frequencies H2.3: FRT acceptance is higher among citizens who have been exposed to higher frequencies of public use
Perceptions		
Consequences	*1* *=* *Convenience, 2* *=* *Privacy violation, 3* *=* *Efficiency, 4* *=* *Discrimination, 5* *=* *Security, 6* *=* *Surveillance, 7* *=* *None of the above*	FRT acceptance is higher among citizens who think FRT will enhance convenience (H3.1), efficiency (H3.2), and security (H3.3). FRT acceptance is lower among citizens who think FRT will enhance privacy violation (H3.4), discrimination (H3.5), and surveillance (H3.6)
Usefulness	*1* *=* *Smartphone usage, 2* *=* *Smart devices and gadgets, 3* *=* *Public streets, 4* *=* *Railway, subway stations, 5* *=* *Customs control or security, 6* *=* *Tourist attractions, 7* *=* *Identity verification for financial matters, 8* *=* *Shopping malls, private shops, 9* *=* *Schools or universities, 10* *=* *Private households, 11* *=* *None of the above*	H3.7: FRT acceptance is higher when citizens perceive the technology to be useful in one or several of the areas/occasions
Reliability	*1* *=* *Less reliable, 2* *=* *Neither more nor less, 3* *=* *More reliable, 99* *=* *Don*’*t know, for regression dummy variable: 0* *=* *Less reliable/Neither more nor less/Don*’*t know, 1* *=* *More reliable*	H3.8: FRT acceptance is higher among citizens who think FRT is more reliable than other identification technologies

FRT: facial recognition technology.

### Antecedent factors: Perceived consequences, usefulness, and reliability

Drawing on the literature of privacy-security trade-off and the TAM and UTAUT models, perceived usefulness and reliability of the technology are antecedent factors that affect how citizens come to accept FRT. Usefulness refers to how useful the technology is in different usage contexts. Perceived consequences of using a technology can include risks and benefits. Perceptions of them can thus be positive in nature, such as increased efficiency, convenience, and security, or negative such as privacy violations, discrimination, and surveillance. Studying public opinion in Western democracies, previous studies mainly focus on citizens’ privacy-security trade-off (Davis and Silver, 2004; Dietrich and Crabtree, 2019; Pavone and Degli Esposti, 2010). The assumption here is that with free media and access to information, citizens understand the risks and benefits associated with FRT and accept that the state violates their individual freedom in exchange for the promise of greater security (Dietrich and Crabtree, 2019). Pavone and Degli Esposti (2010) show in their survey across six European countries that citizens’ assessment of surveillance-oriented security technologies (SOST) is largely based on the relational social context of technology implementation. Those who trust political institutions tend to consider SOSTs as effectively enhancing their security; those who expressed concern about government’s surveillance intentions regard SOSTs as mainly privacy infringing. In authoritarian states, one could assume that there is less room for open discussion and citizens might either be less informed about potential risks due to government information control or feel powerless to oppose them. How exactly citizens in an authoritarian context view the functions and uses of FRT, however, is not fully understood. Wang and Zhang (2019) find that supporters of FRT in China downplay privacy issues by either fundamentally denying them or arguing that the gains of enhanced security outweigh the losses to individuals’ privacy. Yet, in both authoritarian and Western contexts, discussions mainly focus on the privacy-security trade-off, overlooking other functions and uses, such as convenience, efficiency, or surveillance.

**Figure 1.**
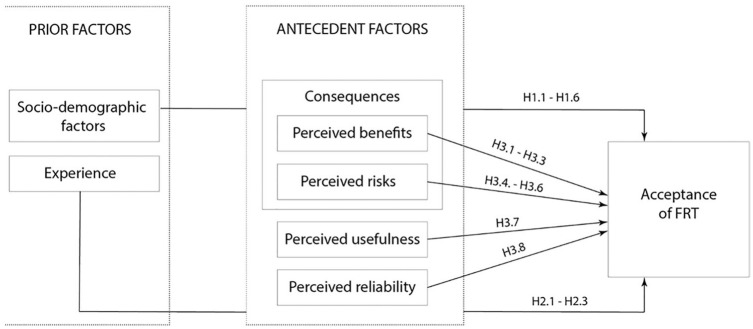
Conceptual framework. Acceptance model integrating priors and perceptions.

Moreover, beliefs about the reliability of a technology potentially influence citizens’ acceptance levels. Depending on the clarity of the image, quality of the matching process, and diversity of the database, numerous flaws in the facial recognition’s accuracy can result in misidentifications, especially when applied to minorities and women (Grother et al., 2018). Knowledge about such inaccuracies might negatively influence public attitudes toward FRT. This suggests that, if aware, minorities and identities shown to more likely be the target of misidentification might also be less likely to accept surveillance technology. Based on these previous findings, we derive the third set of hypotheses for our study: H3.1–H.3.8 (see Table 1). Figure 1 summarizes our conceptual framework of FRT acceptance integrating the aforementioned prior factors and the perceptions illustrated above.

## 3. Methodology

### Data sources and questionnaire design

Between August and September 2019, we conducted an online survey in China, Germany, the United Kingdom, and the United States through a Berlin-based survey firm. As the agency cooperates with app and mobile website providers in each of the four countries, the survey was administered online through mobile applications. As a sampling method, we used “river sampling,” also referred to as intercept sampling or real-time sampling (Lehdonvirta et al., 2020), drawing participants from a base of 1–3 million unique users.^1^

This allowed for both first-time and regular survey-takers to participate. From a network of more than 40,000 participating apps and mobile websites, our survey included respondents through more than 100 apps comprising different formats and topics, such as shopping (e.g. Amazon), photo-sharing (e.g. Instagram), lifestyle (e.g. DesignHome), and messaging (e.g. Line). Offer walls provided options to receive small financial and non-monetary rewards as an incentive to take part in our survey, such as premium content, extra features, vouchers, and PayPal cash. Users did not know the topic of the questionnaire before opting in to participate, thereby minimizing topical self-selection (Lehdonvirta et al., 2020). Instead, each participant underwent a pre-screening before being directed to a survey they were matched to. The conversion rate of users who fully finished the survey was 70% (China), 73% (Germany), 69% (the United Kingdom), and 67% (the United States), respectively. Several consecutive identical answer choices or disproportionately quick completion of a questionnaire prompted invalidation. This method provided us with a sample size of 6633 citizens.

The survey is a non-probability online survey using quota sampling. Sampling quotas were created from the most recent population statistics available from the Barro Lee (2017) Census Population Data and adjusted for the Internet penetration data according to information from the Pew Global Attitudes Survey (2017) for China, and regional population statistics from Statistica (2016). Findings from this online survey thus resemble the *Internet*-connected population in each country—meaning slightly younger and maybe higher technology-affinity than the overall population. The quotas used for sampling and weighting were set on age (18–65) and gender. For China, respondents were also sampled according to region, including quotas for the three main regions of China: Central, (37%) Western (21%), and Eastern (42%). In the other countries, equal attention was paid to ensure accurate representation of local regions, including adequate representation of federal states in Germany, counties in the United Kingdom, and states in the United States. After collecting the necessary number of respondents meeting quotas for each sub-population, a weighting algorithm corrected for any minor discrepancies between the collected sample and the quotas, correcting for under- and over-representation of each group.^2^ The maximum weight allocated was 1.8 and the overall margin of error for estimates is 2.4% for China, 2.4% for Germany, 2.5% for the United Kingdom, and 2.5% for the United States. Supplemental material online offers more information on the survey’s method and summary statistics.

### Data analysis

Responses to the questionnaire were examined using ordered logistics regression analysis.^3^ As we sought to analyze the effects of socio-demographic factors, political context, and citizens’ perceived functions of FRT, our dependent variable of interest is “social acceptance.” The question reads: “In general, do you accept or oppose the use of facial recognition technology?” allowing the responses *strongly oppose, somewhat oppose, neither oppose nor accept, somewhat accept*, or *strongly accept*. Levels of acceptance were investigated by analyzing people’s individual characteristics and familiarity, followed by studying different political context, and their perceptions about the consequences and functions of FRT. Table 1 summarizes the measurements and hypotheses related to our independent variables. Of the 6633 respondents in our sample, 8.1% (*N* = 535) had “never heard about FRT” prior to taking the survey. Given the focus of our study on political context and perceived functions and consequences of FRT, we *excluded* those 8.1% from our analysis which left us with 6099 citizens: 1628 in China, 1538 in Germany, 1524 in the United Kingdom, and 1409 in the United States. As this is an online study in multiple countries, we report data for all respondents as well as by country.

## 4. Results

Overall, the findings show a high level of general awareness about FRT. Of the 6633 respondents in our sample, 92% (6099) had “heard about FRT” prior to taking the survey. Only 12% of respondents had not personally observed FRT being used in a private or public context. Most commonly witnessed is the use of FRT in smartphones (57%), followed by customs and security checks at airports (38%), smart devices and gadgets (37%), and identification verification for financial matters (25%). Smartphone usage rates for FRT are particularly high in China with more than 78%, followed by the United States (59%). Additional information on FRT uses by country is summarized in the supplemental material.

### Social acceptance of FRT

**Figure 2.**
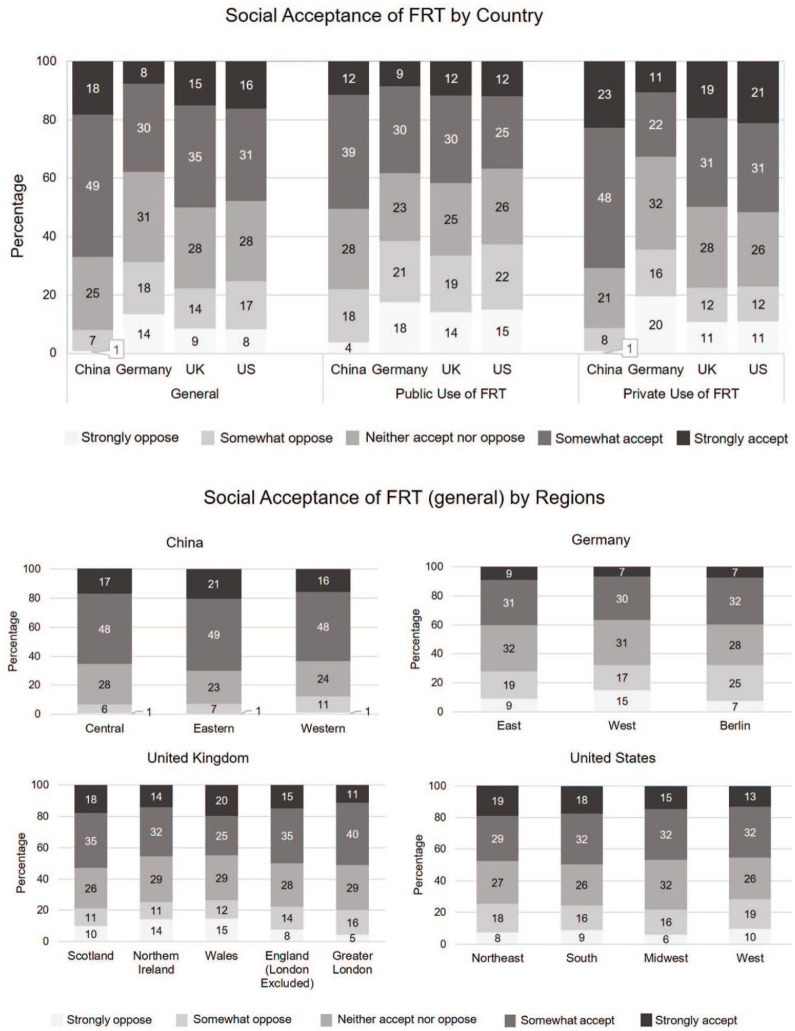
Social acceptance of FRT by country and region. Total *N* = 6099; distributions vary for regions: in China Central: *N* = 601, East: *N* = 686, West: *N* = 341; in Germany, East Germany: *N* = 172, West Germany: *N* = 1228, Berlin: *N* = 139; in the UK, Scotland: *N* = 207, Northern Ireland: *N* = 96, Wales: *N* = 37, England (London excluded): *N* = 1082, Greater London: *N* = 102; in the United States, Northeast: *N* = 269, South: *N* = 556, Midwest: *N* = 317, West: *N* = 268. For the United States, we use Census Data (US Census Bureau, 1995) to divide the states into four regions.

Acceptance rates vary across countries, with 67% of Chinese showing the two highest levels of acceptance, while only 38% of Germans are strongly or somewhat accepting of FRT. This provides support for previous studies arguing that Germans have a higher than normal distrust toward such state surveillance technology applications (Freude and Freude, 2016). The UK and the US responses are in between, with 50% of the UK and 48% of the US respondents expressing acceptance of the technology. All four countries share a similar proportion of respondents with neutral attitudes toward FRT: respectively, 25% of Chinese, 31% of German, and 28% of both the UK and the US participants. Opposition to FRT shows interesting variation in the four countries again: a very low 9% expressed either some or strong opposition to FRT overall in China, while this was much higher with 31% in Germany, 22% in the United Kingdom, and 25% in the United States (see Figure 2).

In Figure 2, we also summarize within-country regional variation in general levels of acceptance. In China, 70% of citizens living in the more economically developed Eastern China somewhat or strongly accept, while this rate is slightly lower for Central China and Western China with 65% and 63%, respectively. In Germany, acceptance seemed slightly higher in the East (former German Democratic Republic (GDR)) with 40% either somewhat or strongly accepting the technology as compared with 37% in the West. Berlin was reported separately as it includes both former West and East and surprisingly has higher acceptance levels of 39%. Interestingly, 15% of respondents in the former West Germany strongly oppose the technology, one of the strongest rejection rates among all regional groups in the sample. This strong opposition parallels the high privacy concerns within this group, with 90% of those respondents stating that FRT poses a threat to privacy. Overall, the responses in East Germany are more positive despite respondents having once experienced state surveillance. In the United Kingdom, the highest acceptance can be found in Scotland with 53% of citizens either strongly or somewhat accepting FRT, followed by Greater London (51%), England (50%), Northern Ireland (46%), and Wales (45%). Opposition to FRT is particularly strong in Northern Ireland and Wales with 14% and 15% respondents, respectively, strongly opposing the technology. Acceptance levels in the United States are high in the Northeast (48%) and South (50%) and lower in the Midwest (47%) and West (45%).

Acceptance rates are even higher when respondents are asked specifically about the *private* use of FRT, as shown in Figure 2. Acceptance is particularly high in China at 71%, second highest in the United States at 52%, followed by the United Kingdom at 50% and Germany at 33%. By comparison, acceptance levels decline but are still high when asked about use of FRT for *government* use with 42% of all respondents accepting it. Acceptance levels are again highest in China at 51%, while in the United States, lowest at only 37%. In the UK sample, 42% accept the FRT software and in Germany 38%.

When asked about the extent to which respondents would accept FRT when managed by central or local governments, private companies, or public–private partnerships (PPPs), a slightly different picture emerges. Here, the acceptance for FRT use by private enterprises is only 15% in Germany, 17% in China, 20% in the United Kingdom, and 30% in the United States. Acceptance for the central government as a provider rises to 60% in China, 47% in the United Kingdom, 37% in Germany, and 35% in the United States. The most preferred choice as a provider seems to be PPPs, with 58% in Germany, 53% in China, 51% in the United Kingdom, and 48% in the United States.^4^ These figures suggest that citizens differentiate between private use of FRT (i.e. using FRT personally/privately is acceptable), while they do not trust commercial companies as providers. There also seems to be limited understanding of the dynamics behind FRT provisions, as almost all state surveillance technology involves a private company providing it.

In addition to questions on acceptance, the survey also asked respondents if they think that FRT generates more risks or benefits. The majority of Chinese citizen (68%) link FRT with more or at least slightly more benefits than risks, a rate much higher than that in Germany (53%), the United Kingdom (54%), and the United States (51%). A similar picture also emerges when asking respondents if they generally support or oppose the use of surveillance by their government: 52% of respondents in the China sample somewhat or strongly support this, compared with 40% in Germany, 47% in the United Kingdom, and 38% in the United States.

### Effects on social acceptance

**Figure 3.**
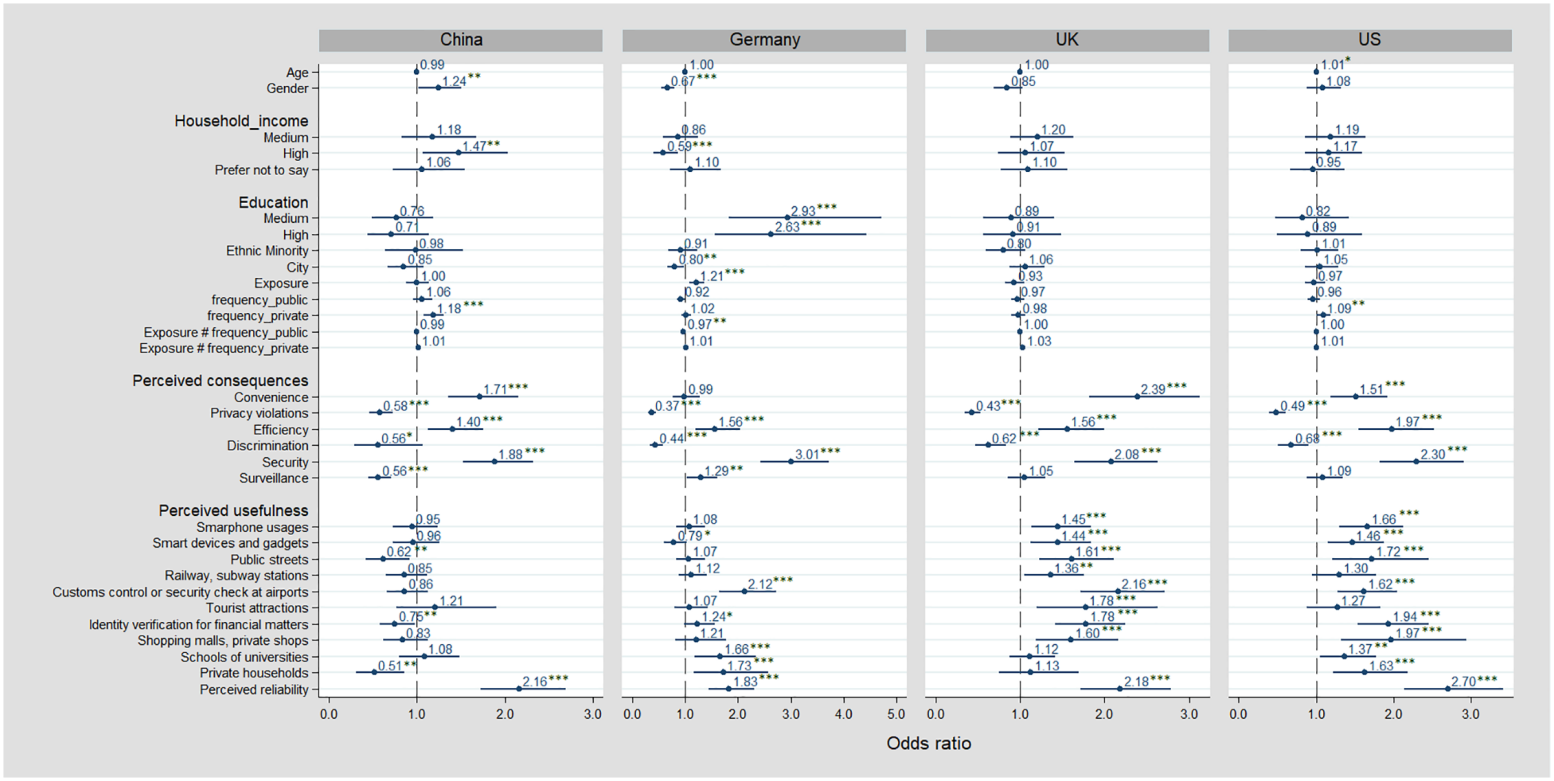
Ordered logistic regression: acceptance of general use of FRT. *p < .10, **p < .05, ***p < .01.

Our hypotheses generated a range of predictor variables related to socio-demographic factors (H1.1, H1.6), experience (H2.1, H2.3), and perceptions of consequences, usefulness, and reliability of FRT (H3.1, H3.8). To assess the power of these variables and examine how they are associated with acceptance of FRT, we undertook an ordered logit regression, summarized in Figure 3. The focus is on FRT use in general, but additional regression analysis on comparing effects on social acceptance of FRT for private and public use is presented in the supplemental material.^5^ Our focus was on respondents who indicated they were aware of FRT (*N* = 6099). Our model measured the effects of socio-demographics and experience as well as perceived consequences, usefulness, and reliability. We ran the regression with this combined factor model for each country.

Our analysis finds that age has a small, positive significant association with FRT acceptance in the UK and US samples; the older a participant, the more likely they are to accept FRT, albeit the effect being very small. For China and Germany, age is not significantly associated with FRT acceptance, suggesting that age is not a key factor to explain variation in acceptance levels, providing no support for H1.1. We also find variation for gender: while it is not significantly associated with FRT acceptance in the United Kingdom and the United States (not providing support for H1.2), FRT acceptance is significantly higher among male citizens in Germany and among female citizens in China. The odds ratios (ORs) for income show a stronger, significant positive effect for the high-income group in the China, the UK and the US samples, suggesting that in these countries, a high income increases likelihood of acceptance (H1.3), though not in Germany.^6^ The finding for Germany is particularly interesting: the odds of accepting FRT is 41% (OR = .59) lower for high-income groups than for low-income groups in this country, holding other factors constant. Possibly, Germans prioritize their privacy or do not have a strong sense of having to rely on FRTs for their safety. The level of education is only positively and significantly associated with FRT acceptance for the German sample, not supporting H1.4 for China, the United Kingdom and the United States. The predictor related to being a member of an ethnic minority is insignificant in all four countries, not supporting H1.5. The regression shows that living in an urban area is only significantly associated with FRT acceptance in Germany (negative association), while for the other three countries, the association is insignificant, thereby not providing support for H1.6 in these contexts.

Another explanatory factor is exposure to FRT use. According to H2.1, exposure to many different uses of FRT is positively associated with one’s acceptance to FRT. The results show that this seems to be only the case for Germany; results for the other three countries are insignificant. In China and the United States, FRT acceptance is higher among citizens who have used FRT privately at higher frequencies, with both strong, positive, and significant effects, providing support for H2.2. However, FRT acceptance is lower among citizens who have been exposed to higher frequencies of public use in Germany, not providing support for H2.3. For the other three country samples, the results remain insignificant.

The regression further shows a very large positive significant association for convenience in each country except Germany, providing support for H3.1. We also found a large, significant, positive effect for efficiency, and security for all four countries, providing support for H3.2 and H3.3. The odds of accepting FRT among citizens who believe FRT enhances security is 1–3 times greater than those who do not share this perception across all four countries (OR = 1.0, 3.01, 2.0, and 2.3 for China, Germany, the United Kingdom, and the United States, respectively), holding other factors constant. The perception of privacy violations (see H3.4) is consistently significantly and negatively associated with FRT acceptance in all four countries. In other words, participants who anticipate privacy violations as a consequence of FRT are less likely to be accepting of it, providing support for H3.4. We also found that the perception of heightened discrimination is significantly and negatively associated with FRT acceptance in all countries again, suggesting that assuming more discrimination decreased those participants’ accepting stance toward the technology, providing support for H3.5. The association of surveillance with FRT acceptance was found significant for China and Germany. In China, anticipated surveillance negatively correlated with acceptance levels, while in Germany, it is positively correlated. This finding is surprising, as we would expect quite the opposite. In the United Kingdom and the United States, surveillance is not significant, providing no support for H3.6 in those settings.

Our model also looked at the perceived usefulness of FRT in a range of areas. For the United Kingdom and the United States, acceptance rates increase among citizens who perceive the technology to be useful in a variety of occasions, such as smartphone usages, smart devices, public streets, and security checks at airports (providing support for H3.7 in these country-specific settings). Finally, findings show that reliability is strongly, significantly, and positively associated with FRT acceptance for each country, providing support for H3.8 in those settings.^7^ The odds for citizens who regard FRT as more reliable than other identification technologies to accept FRT is about two times greater than those who do not share this perception in all four countries (OR = 2.16, 1.83, 2.10, and 2.70 for China, Germany, the United Kingdom, and the United States, respectively), given other factors are held constant.

### Discussion

Our model for acceptance sought to integrate prior factors and perceptions of individuals affecting their stance on FRT. Since initial models of technology acceptance were focused mostly on IT adoption at the workplace and follow-up frameworks were often only applied in national studies for individual countries, this research contributes to the field by being technology-specific but context-agnostic. Using this model, we derive a number of observations about the expanding realm of FRT acceptance studies.

First, the data reveal a range of attitudes about FRT globally. Respondents in China express acceptance levels toward FRT that are almost twice as high as Germans, while respondents in the United Kingdom and the United States are in between. Acceptance levels were especially high for private usage of FRT. While in China, more than 70% of respondents accepted the technology for private use, acceptance drops to 30% in the United Kingdom. Acceptance for public usage is also high, with the highest acceptance rate in China with 50% and lowest rate of 37% in Germany. The findings differ from the Pew Center Survey, which found more acceptance for uses of FRT by public law enforcement agencies rather than companies (2017).

Second, when considering prior factors of individual characteristics and experience levels, the study provides various results across different countries. In general, socio-demographic aspects and exposure to and use of FRT proved to be less statistically significant determinants or if significant, they have a small effect. This contradicts some previous findings related to familiarity increasing technology acceptance (Idemudia and Raisinghani, 2014; Komiak and Benbasat, 2006). Acceptance of FRT is generally higher among the younger, highly educated and higher income population. Such positive attitudes of the highly educated and higher income population are surprising, as for instance previous research by Pan and Xu (2018) suggest that in China the young, better-educated, coastal urban residents lean toward liberal views, and there is an expectation that liberals would be more skeptical of technologies that can be used for surveillance. One possible explanation is that the more educated and higher income group sees FRT through particularly positive frames, such as offering security or convenience, or as a mechanism that affords them personal rewards. In China, the government and state-led media has also repeatedly framed FRT as an instrument to detect corruption by local government officials (Li, 2018). In Germany, high or medium education has one of the strongest positive associations with FRT acceptance, suggesting that more educated citizens are more accepting of FRT. Markedly, this result stands in contrast to the findings from the existing study on public acceptance on surveillance policies in the German population (Trüdinger and Steckermeier, 2017), but matches results from the United States (Fletcher et al., 2017). As the present study was conducted in a context of more news coverage of FRT and its wider spread application, this finding could point to evolving attitudes in Germany.

Investigating the antecedent factors, the results show that perceptions of consequences, usefulness and reliability are largely statistically significant across countries. Perceived risks and concerns for privacy have proven to be decisive for the intention to accept biometric systems (Miltgen et al., 2013) and surveillance technologies (Van Heek et al., 2017). We found that a more comprehensive range of personal judgments are significant factors in the realm of FRT. Although the direction of coefficients at times vary internationally, this overall finding underlines the importance of individual impressions and interpretations of a technological application like FRT as determinants of its acceptance. This in turn signals the gravity of public information and framing of a new technological application, particularly in the case of FRT which offers both voluntary private use and involuntary public exposure.

**Figure 4.**
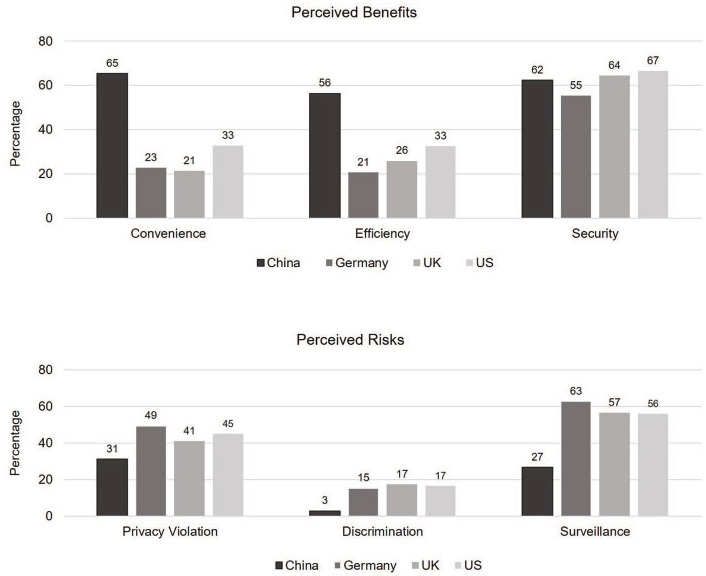
Perceived consequences of FRT by country (*N* = 6099).

More specifically, one interesting finding arises from country differences in perceived consequences of FRT, comprising risks and benefits, shown in Figure 4. Results show that perceiving improved security to be a consequence is a particularly strong, positive factor for explaining FRT acceptance across all countries. Improved efficiency and convenience also appear to be a key factor influencing attitude toward FRT, particularly in China. In other words, Chinese respondents appear to focus on the beneficial aspects of the technology and might thus be considered more techno-optimistic (see Wang and Zhang, 2019). In contrast, in Germany, convenience was not associated with FRT acceptance. This could suggest some skepticism and aversion to techno-utopian narratives of convenience.

Figure 4 shows that a majority of respondents perceive increased surveillance as a consequence though it is only a statistically significant determinant on acceptance in China and Germany. In addition, German respondents particularly fear privacy violations, which in turn, have a strong negative effect on their acceptance. Discriminatory effects of FRT are most noted by the UK citizens and least by Chinese, but overall, the perception of increased discrimination is low, potentially due to differences in media attention to this issue in the United Kingdom and China. A perception of increased security appears to lead to higher acceptance. It is not surprising then that the top three occasions of FRT usage rated most useful are customs control, identification financial matters, and smartphone applications.

### Research limitations

The findings are subject to a number of limitations. First, as this was a non-probability online survey using mobile phones and desktops, the findings can only resemble the Internet-connected population in each country. The results are therefore subject to a “coverage bias,” as subpopulations differ in their access to and use of the Internet (Van Dijk, 2005). Second, while non-probability online surveys offer a fast turnaround time for data collection and are low cost, they are more susceptible to selection biases including topical self-selection and economic self-selection (Lehdonvirta et al., 2020). In this survey, respondents who chose to participate may already have a particular affinity with technology, which could positively affect their stance toward innovations in this field, including the focus of this study. This effect of topical self-selection may have been heightened by the virtual rewards individuals were promised for their participation. This rewards-based recruitment might lead to the selection problem of economic self-selection. Respondents might have been also more likely to associate the positivity of incentives with positivity toward FRT.

Moreover, in China, the authoritarian political context might be reflected in the reported levels of social acceptance, as dissent toward technologies officially endorsed by the government can be difficult. Although participants were aware that any identifying data were anonymized and analyzed for research purposes only, we cannot exclude the possibility of preference falsification as some more cautious respondents may have given false answers due to concerns about reprisals from the state.^8^ For instance, variables such as negative uses of FRT might be underreported among respondents.

Finally, some questions might have also been understood or interpreted differently across countries. As the implementation and use cases of FRT vary widely in the four contexts studied, mentions of the technology may conjure up diverse associations and scenarios. This could influence the connotation participants have when asked about its acceptability. Some questions might also have been misunderstood. For instance, one fifth of the German respondents reported seeing FRT in public streets and railway stations. But given that by 2019, only the train station Berlin Südkreuz had experimented with FRT, and despite the survey’s introductory disclaimer explaining what we mean by “FRT,” some respondents confuse standard video cameras with the more advanced FRT software behind them.^9^ Essentially, unless clearly stated, one cannot know if a simple camera installation is connected to FRT. In addition, our survey likely also contains question biases as offering possible issues or consequences as options may have induced the respondents to report their views (on limited answer possibilities and acquiescence bias, see Furnham, 1986).

## 5. Conclusion

While previous research has identified FRT as an instrument for state surveillance and control, this study shows that surveillance and control are not foremost in the minds of citizens in China, Germany, the United Kingdom, and the United States, but rather notions of convenience and improved security. Based on an online survey resembling the Internet-connected population the study shows high levels of approval for FRT across all four countries. China has the highest citizen approval rates for FRT, Germany the least, with the United Kingdom and the United States in between. Our results illustrate that both prior and antecedent factors of our integrated model help explain international variation in FRT acceptance. In particular, the clear predictive powers of impressions (usefulness, reliability) and anticipations of possible outcomes (risks and benefits) indicate the powerful impact of mental associations, whether based on factual knowledge or inferred perceptions, on FRT’s acceptance.

Implementation of FRT is ongoing on an international scale and it is conceivable that public opinion could shift as FRT software gets more widely adopted. As this study shows, citizens generally trust their government more in the management and provision of the technology than private companies, although more than half of all citizens are also very accepting of public–private partnerships. Given the large cross-country differences in state use of the technology as well as variations in citizens’ acceptance levels, our results raise questions about the feasibility of finding a global regulatory response.

## Supplemental Material

sj-pdf-1-pus-10.1177_09636625211001555 – Supplemental material for Between security and convenience: Facial recognition technology in the eyes of citizens in China, Germany, the United Kingdom, and the United States Click here for additional data file. Supplemental material, sj-pdf-1-pus-10.1177_09636625211001555 for Between security and convenience: Facial recognition technology in the eyes of citizens in China, Germany, the United Kingdom, and the United States by Genia Kostka, Léa Steinacker and Miriam Meckel in Public Understanding of Science

## Notes

1. River-sampling does not include a fixed number of potential survey respondents, as the survey is displayed on offer walls within apps and websites and can, thus, reach millions of users.

2. The current sample is not optimal because given the method of river sampling, active and tech-savvy citizens are also most likely overrepresented in our sample. Post-stratification is a useful tool to improve a sample’s representativeness. However, for questions that we considered to post-stratify, relevant official data are only available at the national level and not concerning geographical, gender, or age distribution. As such, we decided to use weighted data, not to post-stratify.

3. We used a proportional odds model by running ordered logistic regression through Stata ologit program.

4. For more information on respondents’ preferences for facial recognition technology (FRT) providers, please refer to the supplemental material available online.

5. The findings for FRT acceptance in general, private or public use are similar. One main difference is that acceptance for private FRT use is especially high among citizens who have used FRT privately at higher frequencies before.

6. Odds ratios refer to “the odds an outcome will occur given a particular exposure, compared to the odds of the outcome occurring in the absence of that exposure” (Szumilas, 2010: 227). In logistic regression, it is the exponential of the regression coefficient (*e*^b^) and it serves as “an indicator of the change in odds resulting from one unit change in the predicator” (Field et al., 2012: 322).

7. Our variance inflation factors (VIFs) are in an acceptable range from 1 to 5 (if a VIF is > 10, one speaks of having high multicollinearity); see supplemental material.

8. Despite the challenges of conducting public opinion research in authoritarian China, experienced survey researchers argue persuasively that respondents do not systematically falsify their preferences (Tang, 2005).

9. Many “normal-looking” cameras in China actually do have FRT and it is also possible for older cameras to be upgraded to include FRT.
