# Supplementary material for: Between security and convenience: Facial recognition technology in the eyes of citizens in China, Germany, the United Kingdom, and the United States
Source: Public Underst Sci. 2021 Mar 26;30(6):671–90. doi: 10.1177/09636625211001555 (PMC8314996; doi:10.1177/09636625211001555)

Supplemental material

Between security and convenience: Facial recognition technology in the eyes of citizens in China, Germany, the UK and the US

Genia Kostka[a]*, Léa Steinacker[b], and Miriam Meckel[c]

[a]Institute of Chinese Studies, Freie University of Berlin, Berlin, Germany; [b]University of St.Gallen, St.Gallen, Switzerland ,[c]Institute for Media and Communications Management, University of St.Gallen, St.Gallen, Switzerland

*Corresponding author: genia.kostka@fu-berlin.de

Table of Content

Table S1: Summary dependent variable and independent variable

Table S2: Summary statistics by country

Table S3: Model test results

Table S4: Ordered logistic regressions: acceptance of general use of FRT

Table S5: Ordered logistic regressions: acceptance of public use of FRT

Table S6: Ordered logistic regressions: acceptance of private use of FRT

Figure S1: Social acceptance and socio-demographic factors

Figure S2: Use occasions by country

Figure S3: FRT providers - country distribution

Figure S4: Experience - Private use

****

Table S1: Summary Dependent Variable and Independent Variable

	**In general, do you accept or oppose the use of facial recognition technology?** *(weighted, in %)*						
	**Measurement**	*Strongly oppose*	*Somewhat oppose*	*Neither oppose nor accept*	*Somewhat accept*	*Strongly accept*	*N*
			**Dependent variable: Acceptance**				
		7.6	13.7	27.7	36.6	14.3	6099
**Independent variables**			**Sociodemographic**				
**Age**	18-35	6.3	12.1	29.2	37.8	14.5	2788
	36-50	9.1	14.1	26.3	36.3	14.2	2032
	51-65	8.3	16.6	26.8	34.2	14.1	1279
**Gender**	Male	7.5	14.4	24.7	37.5	15.9	3144
	Female	7.8	13	31	35.5	12.6	2955
**Income**	Low	8.3	13.9	33.8	31.0	13.0	798
	Medium	6.1	16.1	27.8	36.0	13.9	2227
	High	9.7	10.8	22.3	40.2	17.1	2318
	Prefer not to say	5.2	15.4	37.8	33.1	8.4	756
**Education**	Low	26.3	10.2	22.9	27.2	13.4	420
	Medium	5.7	14.5	30.5	36.2	13.2	3987
	High	7.5	12.8	22.5	39.8	17.3	1692
**Ethnic Group**	Minority	8	17.1	29	30.8	15.2	866
	Majority	7.6	13	24.6	39.6	15.1	4314
	Don’t know	7.5	13.9	41.3	27.6	9.8	920
**Living area**	Rural	6.1	13.6	30.6	36.7	13	2135
	City	8.5	13.8	26.2	36.5	15	3964
**Exposure to FRT**	0 (None of the instances)	12.2	16.4	35.4	25.4	10.5	744
	1	7.8	13.6	31.7	34.0	12.8	1719
	2	4.9	15.0	28.9	39.0	12.3	992
	3	12.4	11.9	23.4	38.3	14.0	972
	4	3.6	12.9	24.2	42.5	16.7	632
	5	4.6	15.1	20.3	40.6	19.3	467
	6	2.6	12.8	23.2	40.0	21.4	233
	7	5.5	10.8	21.6	45.4	16.7	154
	8	4.4	10.1	16.8	46.1	22.7	62
	9	5.2	10.8	20.1	39.4	24.5	61
	10	8.7	5.2	15.5	44.1	26.5	38
	11	13.7	7.5	22.1	27.2	29.6	26
**Frequency of FRT Private Use**	Never	8.7	18.5	33.7	30.3	8.7	2584
	Several times in my life	2.7	14.1	36.2	37.8	9.2	427
	Several times a year	2.0	13.2	28.5	46.6	9.8	333
	Several times a month	2.4	10.3	32.0	42.0	13.3	383
	Several times a week	2.6	13.0	24.5	45.6	14.4	431
	Most of the time	3.6	12.1	21.4	43.5	19.4	559
	Everyday	13.2	6.6	16.2	38.3	25.7	1383
**Frequency of FRT Public Use**	Never	7.7	12.2	36.6	30.5	12.9	1383
	Several times in my life	4.0	17.5	31.5	38.1	8.8	910
	Several times a year	3.0	14.2	27.5	43.2	12.2	707
	Several times a month	3.0	13.4	27.6	41.0	15.0	652
	Several times a week	5.5	15.7	21.4	42.6	14.8	712

	Most of the time	6.7	13.3	22.6	38.0	19.4	871
	Everyday	21.4	10.7	20.3	29.4	18.2	864
**Perceived functions and accuracy**							
**Conse-quences**	Convenience	6	6.1	20.8	45.2	21.9	2200
	Privacy violations	12.4	22	30	28.6	6.9	2526
	Efficiency	4.7	6.7	19.3	45.9	23.4	2089
	Discrimination	21.1	25	24.7	21.5	7.8	780
	Security	2.8	9.5	25.8	44	18.3	3785
	Surveillance	6.4	16.6	28.5	36.4	12.1	3054
	None of the above	12.1	11.3	42.2	19.6	14.8	265
**Usefulness**	Smartphone usages	6.7	11.1	26	38.8	17.3	2311
	Smart devices & gadgets	7.4	13.2	26.6	37.6	15.2	1894
	Public streets	11.6	12	26	37.1	13.3	981
	Railway, subway stations	6	13	27.7	38.2	15.1	2173
	Customs control or security check at airport	4.3	13.5	29	39.5	13.8	4012
	Tourist attractions	9.2	13.2	29.4	34	14.2	648
	Identity verification for financial matters	3.6	13.8	27.9	40.3	14.4	2532
	Shopping malls or private shops	5	11.4	26.1	39.6	17.8	831
	Schools or universities	5.6	13	30.4	36.6	14.5	1130
	Private household	6.8	19.2	28.2	31.6	14.2	567
	None of the above	32.1	22.8	27	11.4	6.2	406
**Reliability**	Less reliable	20.5	23.5	23.8	27	5.2	898
	Neither less nor more reliable	4.2	14.9	36.5	35.3	9.1	2344
	More reliable	5.2	7.5	17.6	44.4	25.3	2186
	Don’t know	10.6	16.7	35.6	28.1	9	671

Table S2: Summary statistics by country

		Strongly oppose	Somewhat oppose	Neither oppose nor accept	Somewhat accept	Strongly accept
**Social Acceptance of Facial Recognition Technology**						
**Age Group**						
**China**	*18-35*	0.7	6.2	26.0	48.8	18.3
	36-50	0.6	7.4	23.7	50.2	18.2
	51-65	3.3	21.7	18.6	38.1	18.3
**Germany**	*18-35*	14.3	18.0	34.7	27.1	5.8
	36-50	16.3	17.9	27.5	31.0	7.2
	51-65	8.6	17.3	30.6	33.0	10.5
**UK**	*18-35*	6.5	14.8	29.1	35.8	13.8
	36-50	9.4	11.1	27.8	36.1	15.6
	51-65	10.3	15.2	26.0	32.2	16.3
**US**	*18-35*	9.5	15.3	30.5	28.4	16.2
	36-50	7.6	18.5	25.6	30.9	17.5
	51-65	6.8	16.1	25.3	37.2	14.6
**Gender**						
**China**	*Male*	1.1	9.1	23.8	47.5	18.6
	Female	0.5	5.3	26.5	49.9	17.8
**Germany**	*Male*	11.5	17.8	27.0	34.4	9.4
	Female	15.6	17.8	34.9	25.9	5.8
**UK**	*Male*	9.8	13.8	23.9	35.1	17.3
	Female	7.2	13.6	31.7	34.7	12.8
**US**	*Male*	8.5	17.7	24.0	31.5	18.3
	Female	7.8	15.5	31.0	31.4	14.2
**Education Group**						
**China**	*Low*	2.8	7.8	29.0	42.7	17.7
	Medium	0.4	7.4	27.2	48.0	17.0
	High	1.1	7.2	21.1	50.4	20.2
**Germany**	*Low*	52.8	8.4	16.1	14.2	8.3
	Medium	5.9	18.7	34.8	33.6	7.0
	High	21.0	19.6	23.7	26.2	9.6
**UK**	*Low*	14.9	10.5	27.2	34.4	12.1
	Medium	8.1	13.7	29.0	33.9	14.3
	High	8.5	14.4	25.0	37.3	14.7
**US**	*Low*	12.0	18.8	24.2	23.8	21.1
	Medium	8.2	17.2	30.1	30.3	14.3
	High	7.6	15.0	21.5	35.3	20.6
**Income Group**						
**China**	*Low*	0.8	7.7	35.8	40.4	15.3
	Medium	0.8	10.2	25.0	47.1	16.9
	High	1.0	6.1	20.0	52.0	20.9
	Prefer not to say	0.0	7.2	35.8	45.5	11.5
**Germany**	*Low*	11.3	14.3	39.8	27.4	7.3
	Medium	6.0	20.7	33.0	32.1	8.2
	High	25.5	14.4	23.4	28.5	8.2
	Prefer not to say	5.0	20.1	40.6	30.4	3.9
**UK**	*Low*	10.5	17.6	31.0	28.3	12.7
	Medium	8.3	14.0	24.9	37.6	15.3
	High	9.3	10.7	24.2	35.7	20.1
	Prefer not to say	5.8	13.0	42.8	30.0	8.4
**US**	*Low*	12.1	16.5	31.3	26.0	14.2

	Medium	7.0	17.9	27.6	29.6	17.9
	High	7.1	14.3	23.6	36.4	18.6
	Prefer not to say	9.1	20.1	32.9	28.3	9.6
			**Ethnic Group**			
**China**	*Minority*	1.7	6.7	31.1	39.3	21.2
	Majority	0.7	7.5	23.9	49.8	18.2
	Don't know	1.7	6.4	33.9	42.5	15.5
**Germany**	*Minority*	10.0	23.1	29.3	27.9	9.6
	Majority	16.9	17.2	26.6	31.5	7.8
	Don't know	5.3	16.1	45.1	27.7	5.9
**UK**	*Minority*	8.8	15.6	29.8	33.7	12.1
	Majority	8.5	13.4	24.4	37.2	16.6
	Don't know	8.5	13.1	41.0	25.9	11.5
**US**	*Minority*	8.2	17.8	27.5	27.8	18.7
	Majority	7.0	16.7	23.8	35.5	17.1
	Don't know	11.8	15.1	39.8	22.7	10.6
			**Living area**			
**China**	*Rural*	1.1	7.9	26.0	47.9	17.0
	City	0.7	7.1	24.6	48.9	18.8
**Germany**	*Rural*	5.6	18.3	34.4	34.7	7.0
	City	17.4	17.5	29.1	28.0	7.9
**UK**	*Rural*	9.6	13.6	29.5	33.5	13.7
	City	7.8	13.7	26.6	35.8	16.0
**US**	*Rural*	7.4	14.5	32.6	31.5	14.1
	City	8.6	17.9	24.5	31.4	17.5

Table S3: Model test results

			**Linktest**		
Model 1	P>|t|	China	Germany	UK	US
	_hat	0.060	0.000	0.001	0.017
	_hatsq	0.269	0.000	0.635	0.697
Model 2	_hat	0.000	0.000	0.000	0.000
	_hatsq	0.501	0.001	0.584	0.970
			**Mean vif**		
		China	Germany	UK	US
Model 1		4.58	3.31	3.50	3.27
Model 2		2.81	2.26	2.30	2.21

Table S4: Ordered logistic regressions: acceptance of general use of FRT

Ordered Logit Regression, weighted, dependent variable: social acceptance of FRT (general)								
	**Model 1**				**Model 2**			
	**China**	**Germany**	**UK**	**US**	**China**	**Germany**	**UK**	**US**
**Age**	-0.001	0.004	0.006*	0.009**	-0.006	0.003	0.002	0.007*
	(0.005)	(0.004)	(0.004)	(0.004)	(0.006)	(0.004)	(0.004)	(0.004)
**Gender**	0.214**	-0.421***	-0.058	-0.041	0.215**	-0.405***	-0.167	0.076
Female	(0.096)	(0.094)	(0.098)	(0.099)	(0.100)	(0.098)	(0.103)	(0.102)
**Household Income**								
Medium	0.226	-0.078	0.301**	0.338**	0.164	-0.150	0.186	0.170
	(0.167)	(0.183)	(0.146)	(0.160)	(0.179)	(0.190)	(0.156)	(0.166)
High	0.474***	-0.403**	0.364**	0.381**	0.387**	-0.532***	0.065	0.153
	(0.155)	(0.189)	(0.172)	(0.157)	(0.163)	(0.198)	(0.183)	(0.159)
Prefer not to say	0.011	-0.086	0.054	-0.067	0.059	0.097	0.091	-0.046
	(0.179)	(0.204)	(0.163)	(0.175)	(0.193)	(0.214)	(0.179)	(0.184)
**Education**								
Medium	-0.166	1.564***	0.062	-0.112	-0.271	1.076***	-0.115	-0.198
	(0.228)	(0.232)	(0.211)	(0.269)	(0.226)	(0.243)	(0.233)	(0.281)
High	-0.254	1.193***	-0.093	0.003	-0.345	0.968***	-0.092	-0.114
	(0.239)	(0.260)	(0.225)	(0.287)	(0.239)	(0.265)	(0.249)	(0.296)
**Ethnic Minority**	-0.049	-0.010	-0.187	-0.015	-0.015	-0.089	-0.225	0.015
Yes	(0.209)	(0.141)	(0.131)	(0.120)	(0.221)	(0.149)	(0.146)	(0.121)
**City or Rural**	-0.140	-0.234**	0.119	0.017	-0.163	-0.217**	0.060	0.045
City	(0.113)	(0.095)	(0.098)	(0.102)	(0.119)	(0.099)	(0.099)	(0.105)
**Exposure to FRT**	0.003	0.269***	0.096	0.088	-0.003	0.189***	-0.072	-0.028
	(0.065)	(0.054)	(0.059)	(0.063)	(0.066)	(0.059)	(0.065)	(0.068)
**Use Frequency**								
Private	0.186***	0.062	0.035	0.178***	0.169***	0.021	-0.020	0.088**

	(0.046)	(0.039)	(0.043)	(0.040)	(0.048)	(0.042)	(0.043)	(0.042)
Public	0.069	-0.094**	-0.011	-0.062	0.059	-0.085**	-0.034	-0.038
	(0.049)	(0.042)	(0.042)	(0.041)	(0.050)	(0.043)	(0.041)	(0.042)
**exposure # use**	0.019	0.007	0.033**	0.001	0.012	0.012	0.031**	0.005
**freq. private**	(0.012)	(0.012)	(0.014)	(0.012)	(0.012)	(0.012)	(0.013)	(0.013)
**exposure # use**	-0.017	-0.045***	-0.021	-0.010	-0.008	-0.030**	-0.002	0.001
**freq. public**	(0.013)	(0.012)	(0.015)	(0.014)	(0.012)	(0.013)	(0.014)	(0.014)
**Perceived consequences**								
Convenience					0.535***	-0.013	0.870***	0.411***
					(0.117)	(0.131)	(0.138)	(0.124)
Privacy violations					-0.548***	-1.007***	-0.834***	-0.718***
					(0.115)	(0.110)	(0.109)	(0.112)
Efficiency					0.337***	0.445***	0.444***	0.680***
					(0.113)	(0.139)	(0.127)	(0.125)
Discrimination					-0.587*	-0.812***	-0.470***	-0.391***
					(0.330)	(0.143)	(0.144)	(0.146)
Security					0.631***	1.101***	0.731***	0.831***
					(0.106)	(0.109)	(0.120)	(0.120)
Surveillance					-0.574***	0.256**	0.051	0.084
					(0.117)	(0.114)	(0.109)	(0.107)
**Perceived usefulness**								
Smartphone usages					-0.056	0.073	0.368***	0.509***
					(0.135)	(0.126)	(0.124)	(0.126)
Smart devices and gadgets					-0.045	-0.238*	0.366***	0.380***
					(0.139)	(0.135)	(0.125)	(0.125)
Public streets					-0.482**	0.070	0.475***	0.543***
					(0.199)	(0.132)	(0.139)	(0.180)
Railway, subway stations					-0.159	0.114	0.308**	0.261
					(0.137)	(0.117)	(0.130)	(0.160)

Customs control or					-0.152	0.751***	0.768***	0.480***
security check at					(0.138)	(0.129)	(0.117)	(0.120)
airports								
Tourist attractions					0.189	0.071	0.574***	0.240
					(0.231)	(0.145)	(0.199)	(0.185)
Identity verification					-0.288**	0.215*	0.578***	0.660***
for financial matters					(0.136)	(0.120)	(0.117)	(0.120)
Shopping malls,					-0.183	0.190	0.473***	0.676***
private shops					(0.153)	(0.198)	(0.152)	(0.205)
Schools or					0.082	0.510***	0.109	0.312**
universities					(0.160)	(0.173)	(0.122)	(0.134)
Private households					-0.664**	0.546***	0.119	0.488***
					(0.259)	(0.203)	(0.208)	(0.149)
**Perceived reliability**					0.768***	0.605***	0.781***	0.992***
					(0.113)	(0.117)	(0.123)	(0.120)
cut1	-3.628***	-0.961***	-1.596***	-1.555***	-4.047***	-0.857**	-1.117***	-0.417
Constant	(0.476)	(0.333)	(0.334)	(0.380)	(0.569)	(0.391)	(0.384)	(0.432)
cut2	-1.219***	0.273	-0.467	-0.219	-1.510***	0.686*	0.251	1.194***
Constant	(0.372)	(0.336)	(0.328)	(0.367)	(0.475)	(0.387)	(0.383)	(0.426)
cut3	0.588	1.674***	0.830**	1.036***	0.528	2.487***	1.896***	2.771***
Constant	(0.375)	(0.339)	(0.329)	(0.368)	(0.478)	(0.391)	(0.384)	(0.428)
cut4	2.951***	3.766***	2.644***	2.671***	3.217***	4.909***	4.099***	4.744***
Constant	(0.383)	(0.355)	(0.338)	(0.378)	(0.489)	(0.411)	(0.396)	(0.439)

Standard errors in parentheses
* $p < .10$, ** $p < .05$, *** $p < .01$

Note: Dependent variable: social acceptance of FRT (1 = strongly oppose, 2 = somewhat oppose, 3 = neither oppose nor support, 4 = somewhat support, 5 =strongly support). N for Total = 6099, China = 1628, Germany = 1538, UK = 1524, US = 1409.

Table S5: Ordered logistic regressions: acceptance of public use of FRT

	Model 1 Acceptance of FRT (Public)				Model 2 Acceptance (Public)			
	**China**	**Germany**	**UK**	**US**	**China**	**Germany**	**UK**	**US**
**acceptance_frt_public**								
**Age**	0.007	0.009**	0.009**	0.016***	0.003	0.002	0.003	0.012***
	(0.005)	(0.004)	(0.004)	(0.004)	(0.005)	(0.004)	(0.004)	(0.004)
**Gender**	0.207**	-0.206**	0.058	-0.006	0.201**	-0.212**	-0.020	0.116
Female	(0.093)	(0.092)	(0.097)	(0.099)	(0.096)	(0.096)	(0.100)	(0.101)
**Ethnic Minority**	0.232	0.001	-0.118	0.003	0.301	-0.017	-0.176	0.062
Yes	(0.177)	(0.148)	(0.138)	(0.122)	(0.188)	(0.152)	(0.150)	(0.125)
**Education**								
Medium	0.036	1.141***	-0.047	-0.132	-0.079	0.710***	-0.213	-0.144
	(0.226)	(0.208)	(0.190)	(0.271)	(0.250)	(0.219)	(0.222)	(0.293)
High	0.073	0.915***	-0.029	0.108	-0.059	0.703***	0.073	0.023
	(0.235)	(0.237)	(0.205)	(0.288)	(0.261)	(0.247)	(0.237)	(0.307)
**Household Income**								
Medium	0.156	0.173	0.328**	0.187	0.094	0.182	0.208	0.084
	(0.160)	(0.179)	(0.143)	(0.153)	(0.168)	(0.183)	(0.157)	(0.156)
High	0.338**	-0.195	0.223	0.138	0.203	-0.195	0.005	-0.070
	(0.152)	(0.184)	(0.171)	(0.146)	(0.160)	(0.191)	(0.182)	(0.150)
Prefer not to say	0.014	-0.056	0.117	-0.184	-0.021	0.199	0.122	-0.133
	(0.168)	(0.204)	(0.161)	(0.170)	(0.185)	(0.213)	(0.185)	(0.185)
**City or Rural**	-0.003	-0.180*	0.013	-0.039	-0.030	-0.162	-0.063	-0.011
City	(0.109)	(0.098)	(0.099)	(0.104)	(0.112)	(0.101)	(0.100)	(0.106)
**Exposure to FRT**	-0.012	0.292***	0.041	0.028	-0.001	0.219***	-0.094	-0.068
	(0.059)	(0.056)	(0.066)	(0.051)	(0.062)	(0.061)	(0.069)	(0.053)
**Use Frequency**								
private	0.013	-0.081*	-0.037	0.079**	0.020	-0.112**	-0.088**	-0.003
	(0.041)	(0.043)	(0.041)	(0.039)	(0.045)	(0.047)	(0.042)	(0.041)
public	0.098**	0.042	0.012	-0.036	0.090*	0.039	-0.018	-0.017
	(0.044)	(0.045)	(0.042)	(0.041)	(0.047)	(0.048)	(0.041)	(0.041)

**exposure # use freq. private**	0.013	0.026*	0.036**	0.009	0.001	0.030**	0.034**	0.009
	(0.011)	(0.013)	(0.014)	(0.011)	(0.011)	(0.014)	(0.014)	(0.012)
**exposure # use freq. public**	-0.007	-0.063***	-0.015	0.000	-0.001	-0.049***	-0.000	0.012
	(0.011)	(0.013)	(0.017)	(0.011)	(0.011)	(0.015)	(0.016)	(0.012)
**Perceived consequences**								
Convenience					0.683***	-0.150	0.653***	0.539***
					(0.111)	(0.132)	(0.140)	(0.122)
Privacy violations					-0.548***	-1.007***	-1.060***	-0.901***
					(0.117)	(0.109)	(0.113)	(0.115)
Efficiency					0.137	0.368***	0.624***	0.490***
					(0.108)	(0.135)	(0.131)	(0.123)
Discrimination					-0.494*	-0.752***	-0.491***	-0.434***
					(0.295)	(0.145)	(0.142)	(0.155)
Security					0.742***	1.046***	0.581***	0.663***
					(0.102)	(0.107)	(0.117)	(0.118)
Surveillance					-0.428***	0.171	0.004	-0.025
					(0.114)	(0.110)	(0.106)	(0.105)
**Perceived usefulness**								
Smartphone usages					-0.396***	-0.079	0.132	0.216*
					(0.133)	(0.133)	(0.126)	(0.123)
Smart devices and gadgets					-0.103	-0.181	0.192	0.184
					(0.137)	(0.135)	(0.126)	(0.124)
Public streets					0.461**	0.516***	0.781***	0.836***
					(0.207)	(0.135)	(0.135)	(0.178)
Railway, subway stations					0.024	0.490***	0.455***	0.362**
					(0.134)	(0.119)	(0.133)	(0.148)
Customs control or security check at airports					-0.075	0.589***	0.678***	0.395***
					(0.134)	(0.132)	(0.119)	(0.119)
Tourist attractions					0.122	0.170	0.595***	0.424**

					(0.246)	(0.146)	(0.177)	(0.193)
Identity verification for financial matters					-0.501***	0.106	0.355***	0.401***
					(0.134)	(0.125)	(0.115)	(0.118)
Shopping malls, private shops					0.009	0.543***	0.638***	0.689***
					(0.151)	(0.193)	(0.153)	(0.195)
Schools of universities					-0.044	0.642***	0.175	0.290**
					(0.161)	(0.171)	(0.120)	(0.127)
Private households					-0.050	0.306	-0.153	0.328**
					(0.233)	(0.215)	(0.205)	(0.154)
**Perceived reliability**					0.577***	0.457***	0.713***	0.939***
					(0.104)	(0.115)	(0.120)	(0.118)
cut1	-2.138***	-0.342	-1.076***	-0.905**	-2.398***	-0.280	-0.959**	-0.112
Constant	(0.361)	(0.324)	(0.305)	(0.374)	(0.466)	(0.396)	(0.378)	(0.440)
cut2	-0.170	0.849***	0.044	0.326	-0.269	1.227***	0.449	1.372***
Constant	(0.341)	(0.329)	(0.304)	(0.369)	(0.444)	(0.399)	(0.381)	(0.437)
cut3	1.105***	1.858***	1.095***	1.413***	1.192***	2.546***	1.817***	2.727***
Constant	(0.341)	(0.332)	(0.306)	(0.371)	(0.444)	(0.404)	(0.383)	(0.437)
cut4	3.214***	3.843***	2.821***	2.926***	3.551***	4.880***	3.929***	4.515***
Constant	(0.350)	(0.347)	(0.315)	(0.381)	(0.454)	(0.422)	(0.396)	(0.450)

Standard errors in parentheses
* $p < 0.10$, ** $p < 0.05$, *** $p < 0.01$

Table S6: Ordered logistic regressions: acceptance of private use of FRT

	Model 1 Acceptance of FRT (Private)				Model 2 Acceptance of FRT (Private)			
	**China**	**Germany**	**UK**	**US**	**China**	**Germany**	**UK**	**US**
**acceptance_frt_private**								
**Age**	0.007	-0.012***	-0.007*	-0.004	0.005	-0.008**	0.002	-0.002
	(0.005)	(0.004)	(0.004)	(0.004)	(0.006)	(0.004)	(0.004)	(0.005)
**Gender**	0.028	-0.574***	-0.106	-0.097	0.052	-0.452***	-0.167	-0.073
Female	(0.096)	(0.094)	(0.097)	(0.099)	(0.100)	(0.097)	(0.103)	(0.103)
**Ethnic Minority**	-0.029	0.095	-0.272**	-0.037	0.006	0.025	-0.225	0.075
Yes	(0.200)	(0.136)	(0.135)	(0.120)	(0.220)	(0.139)	(0.146)	(0.127)
**Education**								
Medium	-0.013	1.338***	0.125	0.033	-0.048	1.091***	-0.115	-0.029
	(0.234)	(0.218)	(0.201)	(0.241)	(0.242)	(0.227)	(0.233)	(0.249)
High	-0.134	0.924***	-0.126	0.012	-0.074	0.917***	-0.092	-0.057
	(0.250)	(0.254)	(0.215)	(0.258)	(0.257)	(0.263)	(0.249)	(0.267)
**Household Income**								
Medium	0.347**	-0.430**	0.354**	0.460***	0.340*	-0.473**	0.186	0.281*
	(0.168)	(0.187)	(0.142)	(0.154)	(0.174)	(0.191)	(0.156)	(0.161)
High	0.522***	-0.483**	0.566***	0.700***	0.480***	-0.628***	0.065	0.526***
	(0.153)	(0.193)	(0.167)	(0.150)	(0.159)	(0.196)	(0.183)	(0.156)
Prefer not to say	0.255	-0.147	0.120	0.031	0.249	-0.051	0.091	-0.078
	(0.191)	(0.212)	(0.170)	(0.168)	(0.199)	(0.217)	(0.179)	(0.174)
**City or Rural**	-0.063	-0.047	0.072	-0.000	-0.049	-0.065	0.060	0.090
City	(0.117)	(0.097)	(0.097)	(0.104)	(0.120)	(0.100)	(0.099)	(0.107)
**Exposure to FRT**	-0.039	0.141**	0.165**	0.113*	-0.005	0.063	-0.072	0.008
	(0.067)	(0.058)	(0.066)	(0.059)	(0.069)	(0.060)	(0.065)	(0.059)
**Use Frequency**								
private	0.253***	0.185***	0.174***	0.270***	0.236***	0.097**	-0.020	0.157***
	(0.048)	(0.045)	(0.043)	(0.040)	(0.050)	(0.048)	(0.043)	(0.040)
public	0.016	-0.194***	-0.044	-0.088**	0.020	-0.163***	-0.034	-0.053
	(0.049)	(0.043)	(0.041)	(0.041)	(0.052)	(0.047)	(0.041)	(0.041)

**exposure # use freq. private**	0.026**	0.017	0.022	-0.010	0.016	0.026*	0.031**	-0.006
	(0.013)	(0.013)	(0.014)	(0.012)	(0.013)	(0.013)	(0.013)	(0.011)
**exposure # use freq. public**	-0.013	-0.022*	-0.020	-0.006	-0.006	-0.008	-0.002	0.010
	(0.013)	(0.012)	(0.015)	(0.013)	(0.013)	(0.013)	(0.014)	(0.013)
**Perceived consequences**								
Convenience					0.594***	-0.050	0.870***	0.546***
					(0.115)	(0.127)	(0.138)	(0.129)
Privacy violations					-0.567***	-0.911***	-0.834***	-0.530***
					(0.111)	(0.105)	(0.109)	(0.114)
Efficiency					0.043	0.430***	0.444***	0.403***
					(0.111)	(0.131)	(0.127)	(0.130)
Discrimination					-0.514	-0.869***	-0.470***	-0.414***
					(0.366)	(0.160)	(0.144)	(0.150)
Security					0.544***	0.684***	0.731***	0.535***
					(0.108)	(0.106)	(0.120)	(0.122)
Surveillance					-0.432***	0.249**	0.051	-0.031
					(0.113)	(0.109)	(0.109)	(0.110)
**Perceived usefulness**								
Smartphone usages					0.233*	0.777***	0.368***	1.017***
					(0.125)	(0.129)	(0.124)	(0.127)
Smart devices and gadgets					-0.002	-0.160	0.366***	0.825***
					(0.130)	(0.133)	(0.125)	(0.127)
Public streets					-0.339	-0.105	0.475***	0.013
					(0.216)	(0.136)	(0.139)	(0.172)
Railway, subway stations					-0.354***	-0.166	0.308**	-0.133
					(0.130)	(0.122)	(0.130)	(0.151)
Customs control or security check at airports					-0.130	0.315**	0.768***	0.460***
					(0.133)	(0.133)	(0.117)	(0.121)
Tourist attractions					-0.336	-0.345**	0.574***	0.063

					(0.249)	(0.143)	(0.199)	(0.164)
Identity verification for financial matters					-0.458***	0.337***	0.578***	0.364***
					(0.129)	(0.117)	(0.117)	(0.117)
Shopping malls, private shops					-0.277*	-0.117	0.473***	-0.088
					(0.150)	(0.193)	(0.152)	(0.189)
Schools of universities					-0.175	0.174	0.109	0.276**
					(0.163)	(0.165)	(0.122)	(0.124)
Private households					-0.230	0.725***	0.119	0.540***
					(0.299)	(0.210)	(0.208)	(0.152)
**Perceived reliability**					0.643***	0.466***	0.781***	0.786***
					(0.110)	(0.111)	(0.123)	(0.118)
cut1	-3.313***	-1.526***	-1.614***	-1.417***	-3.513***	-1.355***	-1.117***	-0.306
Constant	(0.489)	(0.325)	(0.314)	(0.356)	(0.557)	(0.376)	(0.384)	(0.406)
cut2	-0.630*	-0.621*	-0.703**	-0.507	-0.727	-0.285	0.251	0.771*
Constant	(0.364)	(0.325)	(0.312)	(0.350)	(0.448)	(0.374)	(0.383)	(0.402)
cut3	0.950***	0.866***	0.652**	0.740**	1.005**	1.530***	1.896***	2.308***
Constant	(0.364)	(0.325)	(0.313)	(0.353)	(0.449)	(0.377)	(0.384)	(0.409)
cut4	3.298***	2.462***	2.265***	2.289***	3.626***	3.367***	4.099***	4.167***
Constant	(0.372)	(0.332)	(0.324)	(0.361)	(0.459)	(0.389)	(0.396)	(0.421)

Standard errors in parentheses
* $p < 0.10$, ** $p < 0.05$, *** $p < 0.01$

Figure S1 Social acceptance and socio-demographic factors *(total sample, weighted, in%)*

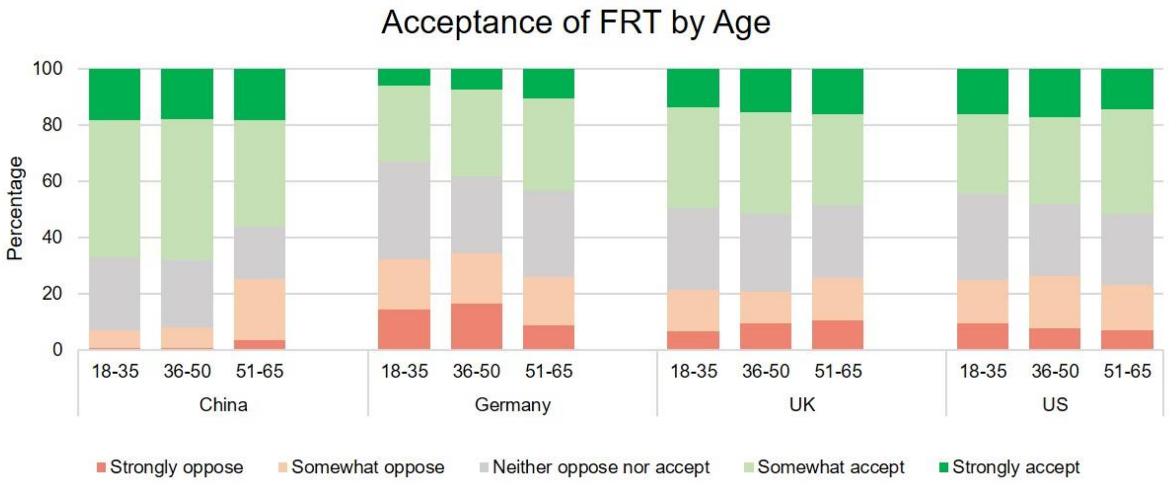

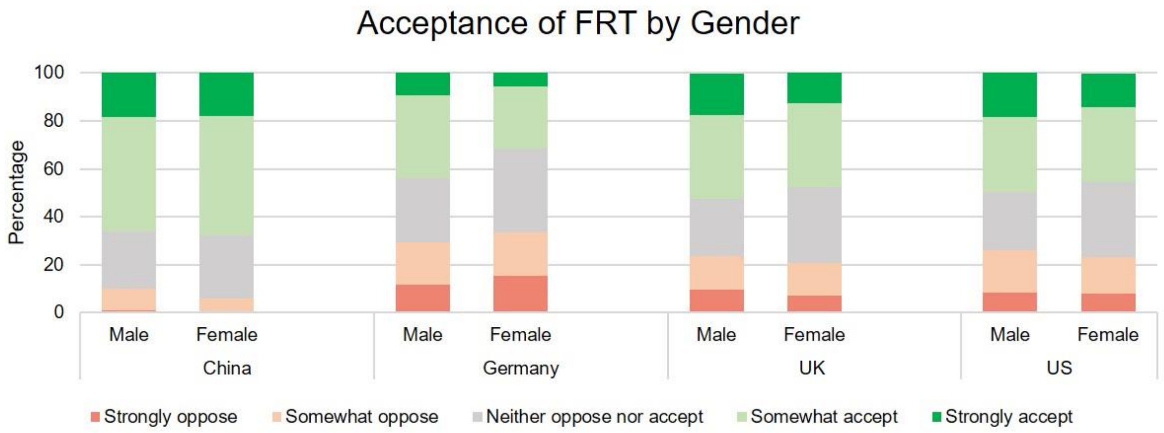

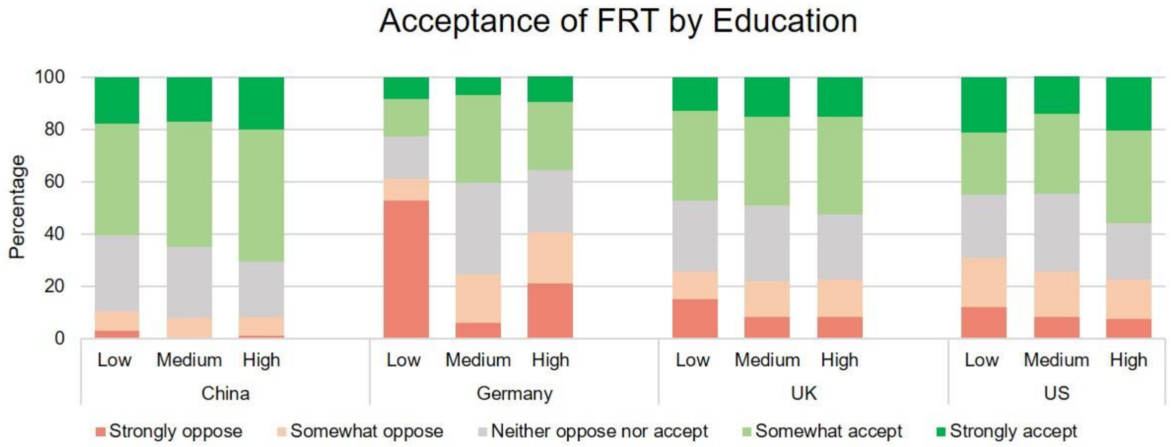

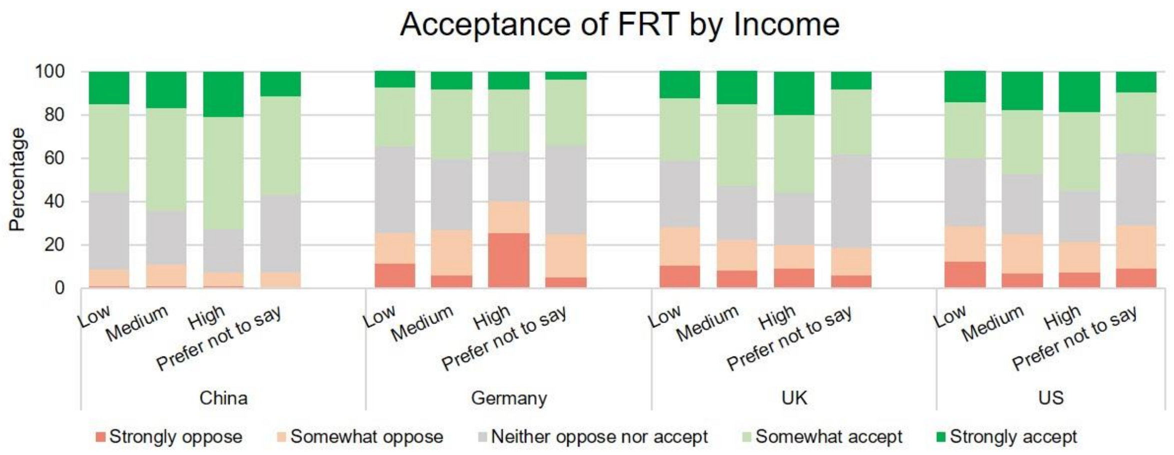

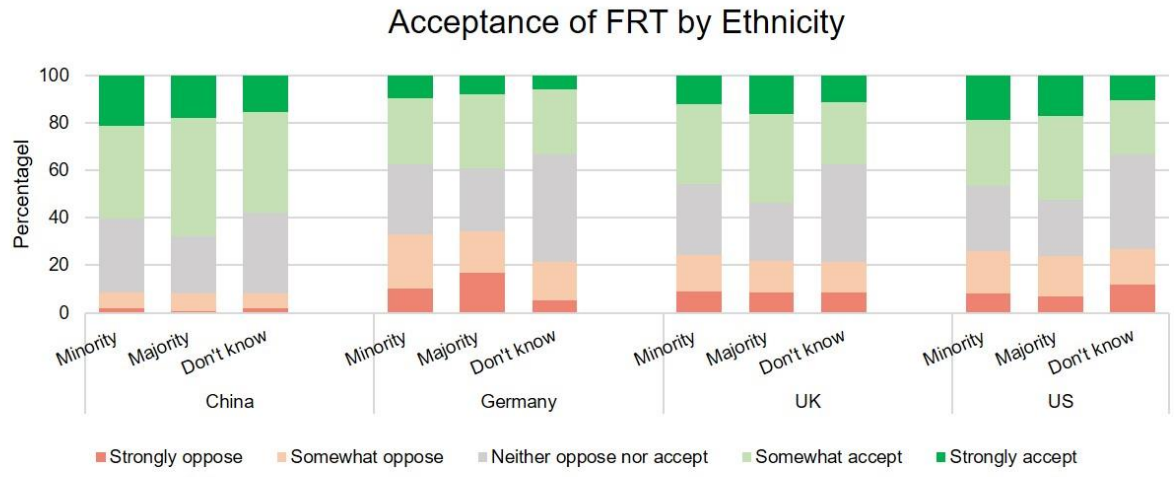

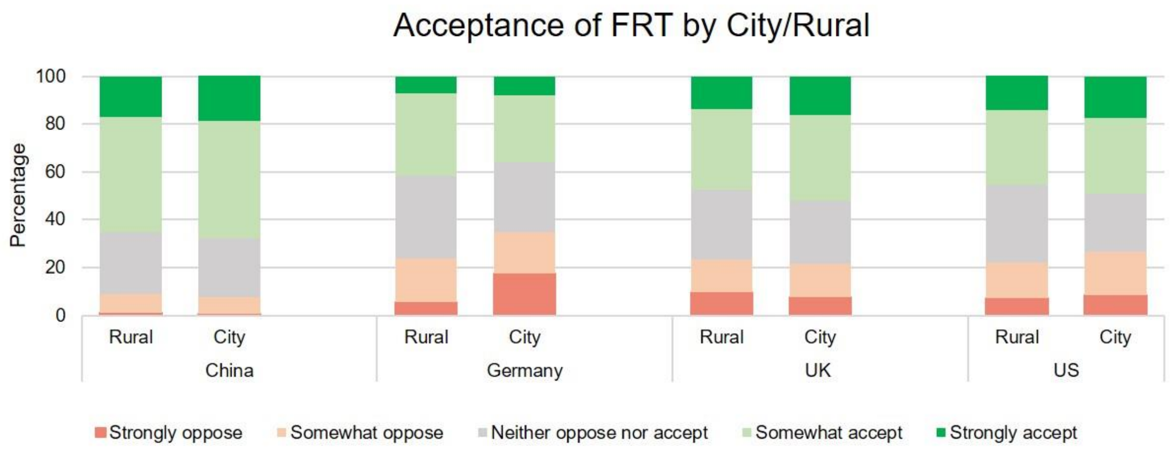

Note: N= 6.099; education was grouped as follows: low (no formal education); medium (high school diploma or equivalent & vocational training); and high (bachelor's degree & master's or doctorate's degree). Income was grouped as follows: *For GER, UK, US: low (under 250-1.000), medium (1.000-4.000), High (above 4.000); prefer not to say; For China: low (under 700-2.100), medium (2.100-5.200), high (above 5.200), prefer not to say; in local currency.*

Table 2: Use occasions by country *(On which of the following occasions have you seen facial recognition technology being used? Respondents answering with yes in %, weighted)*

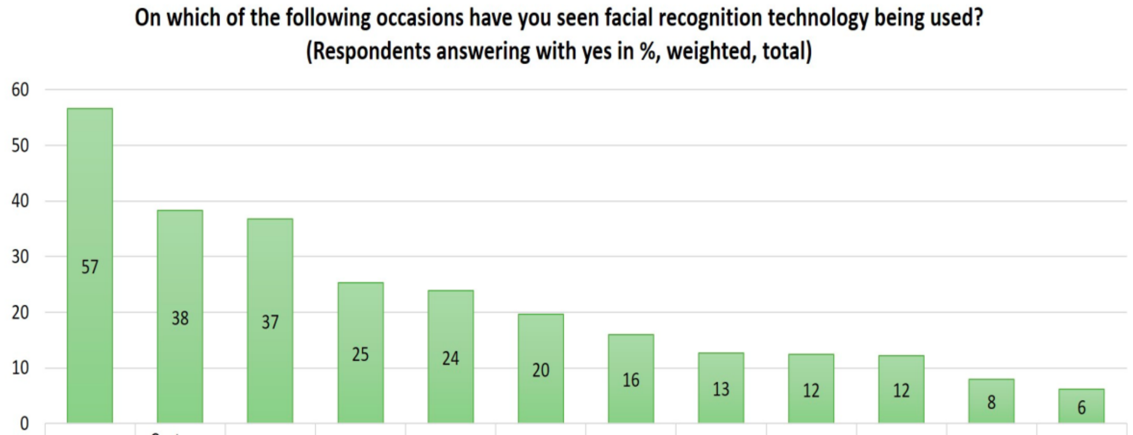

	Smartphone usage	Customs control, security checks at airport	Smart devices & gadgets	Identity verification for financial matters	Railway, subway stations	Shopping malls	Public street	School or universities	Tourist attractions	None of the above	Private household	Others
China	78	42	47	33	45	30	10	21	15	2	8	5
Germany	45	48	18	25	25	17	23	6	12	17	7	8
United Kingdom	44	39	36	19	13	17	18	9	41	18	7	3
United States	59	27	47	23	10	14	14	13	11	14	11	9
Total	57	38	37	25	24	20	16	13	12	12	8	6

Figure S3: FRT Providers - country distribution

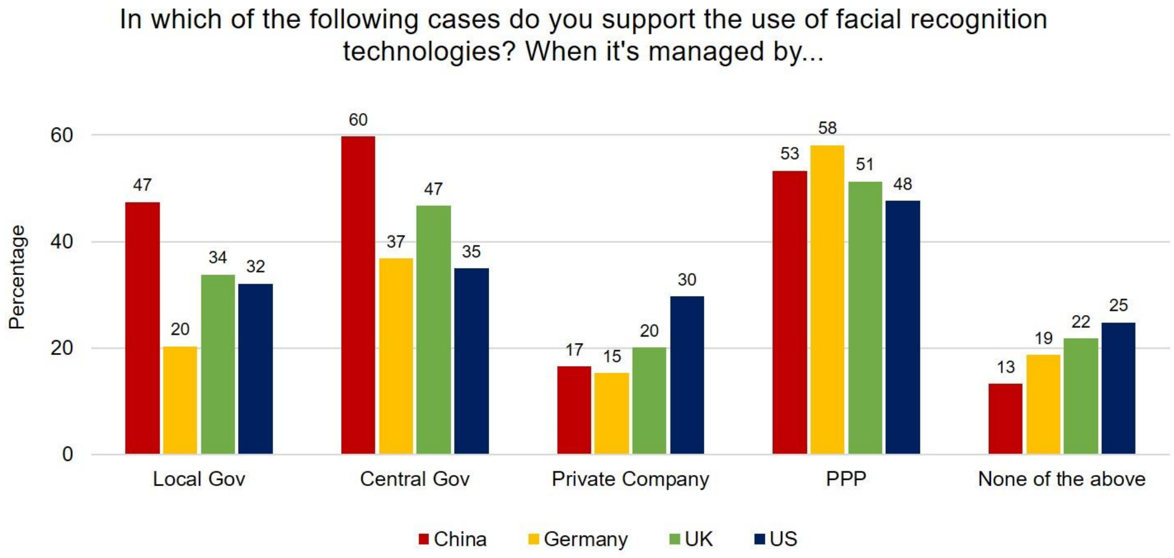

Figure S4: Experience – Private Use

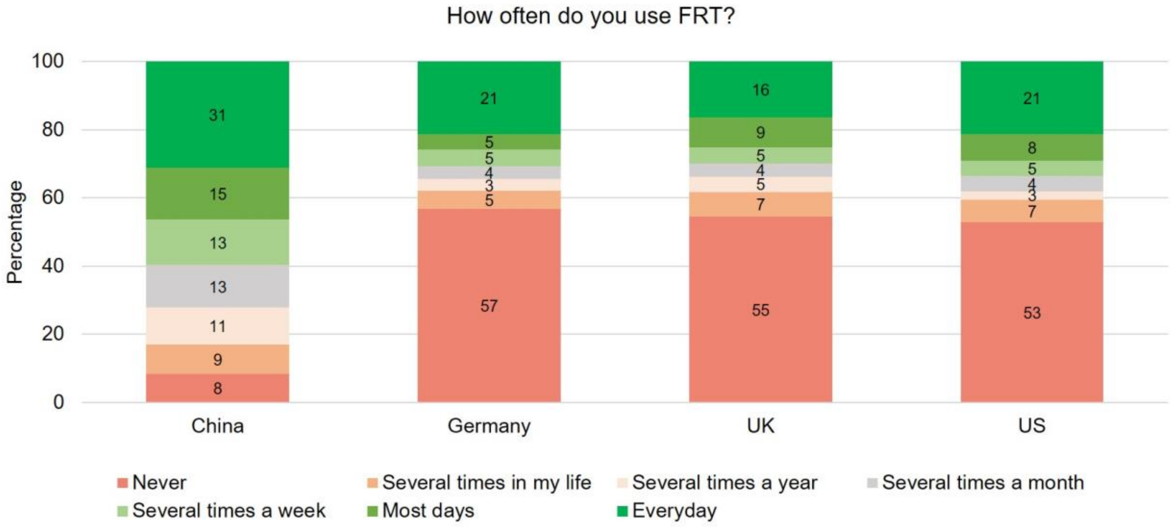

Supplement: sj-pdf-1-pus-10.1177_09636625211001555 – Supplemental material for Between security and convenience: Facial recognition technology in the eyes of citizens in China, Germany, the United Kingdom, and the United States [file sj-pdf-1-pus-10.1177_09636625211001555.pdf]
